# Traditional Use of Medicinal Plants for Symptom Relief During the COVID-19 Pandemic in Bulgaria

**DOI:** 10.3390/plants14233692

**Published:** 2025-12-02

**Authors:** Djeni Cherneva, Nadezhda Nikolova, Tsonka Dimitrova, Dobri Ivanov, Ivelin Iliev, Svetlana Georgieva, Galina Yaneva

**Affiliations:** 1 Department of Biology, Faculty of Pharmacy, Medical University of Varna, 9000 Varna, Bulgaria; n.nikolowa99@gmail.com (N.N.); tsonka.dimitrova@mu-varna.bg (T.D.); d.ivanov@mu-varna.bg (D.I.); galina.yaneva@mu-varna.bg (G.Y.); 2 Department of Pharmaceutical Chemistry, Faculty of Pharmacy, Medical University of Varna, 9000 Varna, Bulgaria; ivelin.iliev@mu-varna.bg (I.I.); svetlana.georgieva@mu-varna.bg (S.G.)

**Keywords:** ethnobotanical study, COVID-19, culturally significant species, ethnobotanical indicators, medicinal plants

## Abstract

The COVID-19 pandemic has renewed global interest in medicinal plants as accessible sources of prophylactic and supportive therapies. Ethnobotanical research provides an important foundation for developing plant-based medicines with preventive and therapeutic potential. This study aimed (1) to investigate the distribution and indications for the use of medicinal plants in the prevention and relief of COVID-19-related symptoms among the Bulgarian population, and (2) to identify culturally significant species with potential for further development as antiviral agents. A total of 513 respondents from different regions and demographic groups in Bulgaria were interviewed. Their knowledge regarding the use of medicinal plants for COVID-19 prevention or treatment was quantitatively assessed using ethnobotanical indices: relative frequency of citation (RFC), informant consensus factor (FIC), fidelity level (FL), and use value (UV). Participants reported 45 species belonging to 43 genera and 23 families. The highest RFC and UVs were recorded for *Matricaria chamomilla* L., *Tilia* sp., *Thymus vulgaris* L., *Zingiber officinale* Roscoe, *Mentha* sp., *Citrus x limon* (L.) Osbeck, *Rosa canina* L., and *Sideritis scardica* Griseb. Culturally significant species identified were *Thymus vulgaris* L., *Matricaria chamomilla* L., *Tilia* sp., *Mentha* sp., *Sideritis scardica* Griseb, *Zingiber officinale* Roscoe, and *Citrus x limon* (L.) Osbeck. This ethnobotanical survey in Bulgaria documents culturally important medicinal plants that may have potential applications in prophylaxis and complementary therapy for COVID-19.

## 1. Introduction

In response to the COVID-19 pandemic, the global scientific community—physicians, pharmacists, biologists—united in the search for effective infection treatment, as well as in finding methods for prophylaxis and prevention.

The efforts of the global scientific community were directed in two main directions: creating vaccines to control the pandemic while simultaneously searching for and creating effective medicines for prevention, treatment and prophylaxis of post-COVID symptoms. In this regard, the integration of traditional and conventional medicine is crucial to achieve effective improvement of symptoms and to lay the foundation for an alternative approach to COVID-19 treatment in the future [1].

The pandemic situation outlined the need for ethnobotanists to adapt to the new environment and conduct ethnobotanical studies to complement the efforts of the medical and pharmaceutical communities in dealing with COVID-19.

Ethnobotanical studies play an important role in preserving traditional knowledge about medicinal plants and their use. The collection and compilation of unrecorded knowledge, transmitted orally through generations, serve as a starting point for modern pharmacognostic and pharmacological research and are, therefore, valuable for pharmaceutical practice [2,3,4,5].

In addition, ethnobotanical studies provide easy access to potential plant sources of bioactive compounds with potential therapeutic application [6].

Today, when the world faces global viral threats, including COVID-19, conducting new ethnobotanical research can serve as a platform for developing effective plant-based medicines for the treatment and prevention of viral diseases [7].

Documenting the folk knowledge of local communities about potential medicinal plants traditionally used for respiratory and viral infections is the first important step in this process.

This motivated the conduct of an ethnobotanical study among the Bulgarian population, which aimed to investigate the use of medicinal plants for the relief of symptoms similar to those associated with COVID-19 and to identify culturally significant species with potential for development as antiviral agents.

## 2. Results and Discussion

### 2.1. Plant Parts, Form of Application, and Procurement Method

#### 2.1.1. Plant Parts

Ethnobotanical analysis of reported phytotherapeutic practices reveals diverse use of various plant substances.

Depending on the symptomatology and etiology of the disease, participants reported the use of various plant parts (root, herb, leaves, flowers, fruits, etc.) in multiple forms of preparation (infusions, tinctures, syrups, essential oils, etc.).

Quantitative analysis of the distribution by type of plant parts demonstrates a significant predominance of reports on the use of flowers (38.05%) and aerial parts (24.39%), followed by those concerning the use of leaves (14.51%), roots (10.98%), and fruits (8.66%) (Table 1).

#### 2.1.2. Forms of Application

Regarding the methodology of preparation and application, data indicate dominance of traditional extraction methods. Decoction constitutes 48.05% (n = 394) of registered cases, infusion—25.85% (n = 212), while application in fresh state was reported by 14.88% (n = 122) of respondents (Table 2).

Some informants report independent use of a medicinal species, while others apply a combination of several. The addition of honey to the applied herbal treatment is very often reported, whose medicinal benefits are documented in numerous studies [8,9].

#### 2.1.3. Procurement Method

Ethnobotanical analysis of medicinal plant procurement practices demonstrates substantial dependence on wild-growing populations.

The dominant source is collection from natural habitats—41.46% (n = 340), followed by commercial networks (markets)—36.71% (n = 301). Procurement from pharmacies and home cultivation represent 19.39% (n = 159) and 2.44% (n = 20) of the total sample, respectively (Table 3).

The observed distribution reflects the sustainability of traditional ethnobotanical knowledge of the Bulgarian population while simultaneously demonstrating the influence of urbanization processes and socio-demographic transformations [10]. Rural–urban migration flows, the dynamics of everyday life, and reduced free time for contact with the natural environment modulate contemporary patterns of medicinal plant use.

### 2.2. Sources of Information Regarding Medicinal Plant Use Specified in the Survey

The main sources of information regarding the use of medicinal plants for prevention and/or treatment of COVID-19, identified in the present study, include oral tradition (elderly people, relatives and acquaintances), specialized literature, internet and media sources, and health professionals, as well as personal practical experience (Table 4).

Data from Table 4 show that in 60.86% of reports (n = 499), respondents report acquiring ethnopharmacological knowledge through oral tradition—from elderly people (33.54%, n = 275) and relatives and acquaintances (27.32%, n = 224).

Specialized literature (books and magazines) is indicated in 16.95% of reports (n = 139), followed by internet and media sources (15.85%, n = 130). Health professionals are indicated least frequently—in 4.51% of reports (n = 37), while personal experience is noted in a minimal number of cases (1.83%, n = 15).

The obtained results demonstrate the dominant role of oral tradition (60.86%) as the main channel for transmission of ethnopharmacological knowledge among the Bulgarian population under pandemic crisis conditions. This pattern of knowledge transmission is characteristic of other cultures with a long-standing use of medicinal plants [10,11,12,13,14,15].

The established high degree of trust in traditional folk knowledge about medicinal plants transmitted orally from generation to generation, which contrasts with the relatively low role of health professionals (4.51%) as a source of information. This imbalance is particularly indicative in the context of a health crisis, when increased recourse to professional medical advice would be expected.

Comparison with ethnobotanical studies conducted in Bulgaria over the last decade shows interesting dynamics: despite observed trends toward the gradual decline of verbally transmitted information flow in favor of modern mass media sources [5], the present study registers the stability of oral tradition under pandemic conditions. This can be interpreted as an actualization of traditional knowledge models in crisis situations.

### 2.3. Ethnobotanical Analysis of the Medicinal Plants Reported in the Survey

Participants in the ethnobotanical study reported the use of 45 medicinal plant species used for COVID-19 prevention and treatment, belonging to 43 genera and 23 families. The best-represented families in terms of the number of medicinal species are Lamiaceae, Asteraceae and Rosaceae. Among them, the Lamiaceae family (122 use reports out of a total of 149) stands out with the greatest contribution to the traditional phytotherapy of the Bulgarian population in the fight against COVID-19.

The consensus among informants regarding the use of medicinal species reported in the study for COVID-19 prevention and treatment was quantitatively assessed using RFC [16,17].

#### 2.3.1. Relative Frequency of Citation (RFC) and Use Value (UV) Analysis of the Medicinal Plants

The RFC values of the medicinal plants mentioned in the survey range from 0.0052 to 0.4124 (Table 5).

The highest RFC value is distinguished by *Matricaria chamomilla* L. (0.4124), followed by *Tilia* sp. (0.3711), *Thymus vulgaris* L. (0.3041) and *Zingiber officinale* Roscoe (0.2475). Species with medium RFC values are *Mentha* sp. (0.2011), *Citrus x limon* (L.) Osbeck (0.1495), *Rosa canina* L. (0.1289) and *Sideritis scardica* Griseb (0.1237), followed by *Origanum vulgare* L. (0.0464), *Melissa officinalis* L. (0.0412), *Hypericum perforatum* L. (0.0360) and *Salvia officinalis* L. (0.0310).

The wide popularity of the mentioned medicinal plants among respondents has its deep roots in the centuries-old experience of the Bulgarian people in the knowledge of herbs and their use, passed down from generation to generation.

With the exception of *Zingiber officinale* Roscoe and *Citrus x limon* (L.) Osbeck, the remaining ten medicinal species—*Matricaria chamomilla* L., *Tilia* sp., *Thymus vulgaris* L., *Mentha* sp., *Rosa canina* L., *Sideritis scardica* Griseb, *Origanum vulgare* L., *Melissa officinalis* L., *Hypericum perforatum* L. and *Salvia officinalis* L. are among the most popular and most frequently used medicinal plants by the Bulgarian population [5,18,19,20,21,22].

Along with *Zingiber officinale* Roscoe and *Citrus x limon* (L.) Osbeck, study participants reported the use of seven more foreign medicinal species for Bulgaria (*Aronia melanocarpa* (Michx.) Elliott, *Hibiscus sabdariffa* L, *Cinnamomum verum* J.Presl, *Laurus nobilis* L., *Armoracia rusticana* G. Gaertn., B. Mey. & Scherb., *Calendula officinalis* L., *Echinacea purpurea* (L.) Moench and *Tagetes erecta* L., which have low RFC values—between 0.0052 and 0.0206.

The reasons for the small number of use reports for the listed foreign medicinal plants may be of different natures (poor awareness of respondents, size of the studied sample, shortcomings related to online interview conduct, etc.), which does not exclude their potential in the fight against COVID-19.

The potential of the foreign medicinal species cited in the study should not be underestimated, as they may also prove useful in the search for active substances for creating new antiviral drugs.

In support of this assumption are numerous scientific data and studies, as well as a number of ethnobotanical studies conducted in different parts of the world, which report their wide traditional use and popularity during the COVID pandemic [23,24,25,26,27,28,29,30].

On the other hand, the reported use of foreign medicinal plants is evidence of the continuity and influence of foreign cultures on the Bulgarian people’s folk knowledge of herbs and their use. We live in a global time in which the boundaries between different peoples and ethnicities are increasingly blurring. The unification of traditional knowledge and experiences of different cultures from around the world in the name of preserving human health and the survival of humanity is of paramount importance.

The effectiveness of herbal treatment applied among respondents was assessed through the UV index, which reflects the degree of distribution of use of a given medicinal species among respondents [31].

According to the reported use of medicinal plants for COVID-19 prevention and treatment in the surveys, the highest UVs are for *Matricaria chamomilla* L. (0.75), *Tilia* sp. (0.72), *Thymus vulgaris* L. (0.55), *Zingiber officinale* Roscoe (0.44), *Mentha* sp. (0.37) and *Sideritis scardica* Griseb. (0.20) (Table 5).

For the remaining plant species, UVs range from 0.005 to 0.14.

The obtained UV data show that the most popular species with widespread use among study participants are *Matricaria chamomilla* L. (0.75), *Tilia* sp. (0.72), *Thymus vulgaris* L. (0.55) and *Zingiber officinale* Roscoe (0.44).

#### 2.3.2. Use Categories and Informant Consensus Factor (FIC)

Participants in the ethnobotanical study indicated one or more uses of the medicinal plants they cited for various symptoms accompanying COVID-19, as well as for disease prevention and prophylaxis.

Based on the data indicated in the survey, a total of 13 use categories were distinguished (“Cough”, “Sore throat”, “Runny nose and loss of smell”, “Respiratory problems”, “Headache”, “Fever”, “Antiviral agent”, “Immunostimulant”, “Sedative”, “Fatigue”, “Treatment of overall symptoms”, “Prophylaxis”, “Prevention of complications”), which can serve as a reflection of the traditional concept of the Bulgarian population regarding the prevention and treatment of symptoms similar to those associated with COVID-19.

The largest share of reports from surveyed participants (23.11%) relates to the use of medicinal plants for relieving the cough symptom. Second are reports for the category “Prevention of complications” (20.55%), followed by the use category concerning sore throat relief (12.35%).

For each category, ethnobotanical data were quantitatively assessed by summing the reported medicinal species, summing individual use reports, and ranking according to the calculated FIC (Table 6).

**Figure 1:**
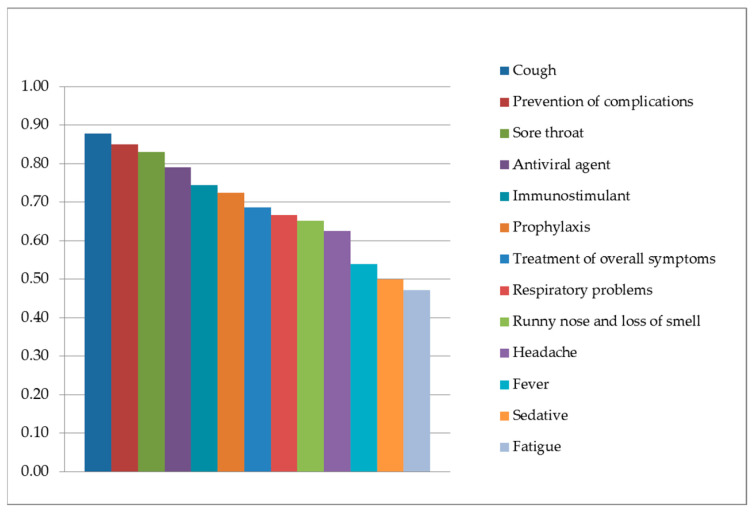
Informant consensus factor for each use category.

The obtained results show a high level of informant consensus for most use categories (average FIC 0.6888) (Figure 1).

The categories with the highest informant consensus (FIC) are “Cough” (FIC 0.8777), “Prevention of complications” (FIC 0.8503), “Sore throat” (FIC 0.8300), “Antiviral agent” (FIC 0.7895), “Immunostimulant” (FIC 0.7436) and “Prophylaxis” (FIC 0.7241) (Table 6).

The high FIC values of the listed use categories show that the ethnobotanical sample is large enough to define a selection of medicinal species that emerge as culturally important in the search for new phytopharmaceutical approaches for COVID-19 prevention and treatment.

The most important medicinal species that determine the high FIC values in the respective use categories are as follows

1. Category “Cough”—*Tilia* sp. (34 reports), *Thymus vulgaris* L. (32 reports), *Matricaria chamomilla* L. (28 reports);
2. Category “Prevention of complications”—*Tilia* sp. (34 reports), *Thymus vulgaris* L. (21 reports), *Zingiber officinale* Roscoe (18 reports);
3. Category “Sore throat”—*Tilia* sp. (28 reports), *Matricaria chamomilla* L. (23 reports), *Thymus vulgaris* L. (14 reports);
4. Category “Antiviral agent”—*Thymus vulgaris* L. (8 reports), *Tilia* sp. (7 reports), *Zingiber officinale* Roscoe (6 reports), *Matricaria chamomilla* L. (6 reports);
5. Category “Immunostimulant”—*Zingiber officinale* Roscoe (12 reports), *Citrus x limon* (L.) Osbeck (9 reports), *Rosa canina* L. (9 reports), *Tilia* sp. (9 reports), *Thymus vugaris* L. (7 reports), *Matricaria chamomilla* L. (6 reports), *Mentha* sp. (6 reports);
6. Category “Prophylaxis”—*Zingiber officinale* Roscoe (2 reports), *Thymus vulgaris* L. (7 reports), *Citrus x limon* (L.) Osbeck (6 reports), *Mentha* sp. (6), *Matricaria chamomilla* L. (5 reports), *Sideritis scardica* Griseb (5 reports);
7. Category “Treatment of overall symptoms”—*Matricaria chamomilla* L. (15 reports), *Mentha* sp. (9 reports), *Thymus vulgaris* L. (8 reports), *Tilia* sp. (7 reports), *Zingiber officinale* Roscoe (5 reports), *Sideritis scardica* Griseb (5 reports), *Clinopodium vulgare* L. (2 reports), *Plantago* sp. (2 reports);
8. Category “Respiratory problems”—*Thymus vulgaris* L. (8 reports), *Matricaria chamomilla* L. (6 reports), *Mentha* sp. (5 reports), *Zingiber officinale* Roscoe (5 reports), *Sideritis scardica* Griseb (4 reports), *Tilia* sp. (3 reports);
9. Category “Runny nose and loss of smell”—*Matricaria chamomilla* L. (9 reports), *Mehtha* sp. (4 reports), *Thymus vulgaris* L. (3 reports), *Tilia* sp. (3 reports);
10. Category “Headache”—*Mehtha* sp. (4 reports), *Matricaria chamomilla* L. (3 reports);
11. Category “Fever”—*Tilia* sp. (5 reports), *Matricaria chamomilla* L. (4 reports);
12. Category “Sedative”—*Matricaria chamomilla* L., (3 reports), *Tilia* sp. (2 reports);
13. Category “Fatigue”—*Rosa canina* L. (3 reports), *Tilia* sp. (3 reports), *Matricaria chamomilla* L., (2 reports), *Hypericum perforatum* L. (2 reports).

The comparative analysis and the high FIC values together indicate that among the study participants, *Matricaria chamomilla* L. is the most widely utilized species with diverse applications, occurring in 12 of the 13 identified use categories. This is followed by *Tilia* sp., cited in 11 categories, *Thymus vulgaris* L., cited in nine categories, and *Zingiber officinale* Roscoe, cited in seven categories. The high FIC values for the main use categories—such as “Cough” (FIC 0.8777), “Prevention of complications” (FIC 0.8503), “Sore throat” (FIC 0.8300), “Antiviral agent” (FIC 0.7895), “Immunostimulant” (FIC 0.7436), and “Prophylaxis” (FIC 0.7241)—demonstrate a strong internal consensus among informants regarding the effectiveness of these species. Lower FIC values in categories such as “Fever,” “Sedative”, and “Fatigue” (around 0.50) do not necessarily indicate low efficacy but rather reflect greater diversity in species usage and potential regional differences in traditional practices [30,32].

The aggregated data analysis shows that *Thymus vulgaris* L., *Matricaria chamomilla* L., *Tilia* sp., and *Zingiber officinale* Roscoe stand out as species with widespread, diverse use, both for relieving disease-related symptoms and for immunostimulation and body prophylaxis.

The large number of reports for their use in different symptom categories speaks both to the stability of folk knowledge and to the stability of traditional healing practices related to the treatment of respiratory and infectious diseases, passed down through generations among local communities of the Bulgarian population.

On the other hand, the relatively small number of species (8 out of a total of 45 reported)—*Thymus vulgaris* L., *Matricaria chamomilla* L., *Tilia* sp., *Rosa canina* L., *Mentha* sp., *Sideritis scardica* Griseb., *Zingiber officinale* Roscoe, and *Citrus x limon* (L.) Osbeck, which have the highest FIC values in all categories—indicates a strongly expressed internal consensus among respondents regarding their effectiveness in the prevention and relief of symptoms associated with COVID-19. This again confirms the stability of traditional phytomedicine practiced for centuries by the Bulgarian population. This is confirmed by a number of ethnobotanical studies that document differences in folk knowledge about medicinal plants and local healing practices among the population in different regions of Bulgaria [5,33,34,35,36].

#### 2.3.3. Fidelity Level Index (FL)—Culturally Significant Species by Use Categories

FL values were calculated to assess how specific the use of medicinal plants indicated in the surveys is for alleviating symptoms accompanying COVID-19.

Since the values of this indicator show specificity but not popularity of a given use of a medicinal species among informants, the sociocultural significance of the medicinal plants mentioned in the study was quantitatively assessed through combined analysis of FL and RFC indices. This approach allows accounting for both the degree of consensus regarding specific use of the species and the proportion of respondents who mention it as particularly important [37].

Plants used by a large number of informants for the same use category are considered culturally significant, while species cited as useful by only one or two informants are considered of low cultural significance [38].

By use categories (with FIC > 0.70), we made a selection of medicinal plants that show high FL value, supported by a sufficient number of informants (more than six). The obtained data are summarized in Table 7.

In the “Cough” category, *Thymus vulgaris* L. and *Tilia* sp. stand out with the highest FL and RFC values, which shows that these species possess high cultural significance and an established role in folk medicine for cough treatment.

*Matricaria chamomilla* L. and *Mentha* sp. have lower FL but high RFC, which indicates that they are widely known, but their traditional use is not limited to cough treatment alone, but also to many other symptoms.

Although a small portion of respondents mention *Sideritis scardica* Griseb. (RFC 0.12), its high FL value (42%) shows that its primary use for cough treatment is established but limited local healing practice.

Although a foreign species to Bulgarian flora, *Zingiber officinale* Roscoe shows a high FL value (37%), which testifies to its successful integration as a foreign species into the traditional healing practices of Bulgarians.

The greatest cultural significance in the “Prevention of complications” category belongs to *Tilia* sp., followed by *Sideritis scardica* Griseb., which, despite lower frequency of mention, shows specific cultural significance among respondents within this use category. *Matricaria chamomilla* L. and *Mentha* sp. are distinguished by great popularity but with lower specificity of use, probably due to their diverse applications in folk medicine. The relatively low FL of *Mentha* sp., despite its wide popularity among respondents, shows that it has universal application and is not specifically associated with a single medicinal use.

In the “Sore throat” category, *Tilia* sp. stands out with high cultural significance and well-defined traditional use. *Thymus vulgaris* L. has medium FL and RFC values, which places it in the category medicinal plants that are traditionally used but limited to several applications, while *Matricaria chamomilla* L. shows high popularity and universal use.

In the “Immunostimulant” and “Prophylaxis” categories, *Zingiber officinale* Roscoe has the largest number of use reports (12 in each), while all other medicinal species are represented with less than ten reports. *Zingiber officinale* Roscoe shows moderate cultural significance and is established as a very well-integrated foreign medicinal species in the modern traditional medicine of Bulgarians, with well-defined medicinal application.

In both categories, *Citrus x limon* (L.) Osbeck shows specific cultural significance, and *Thymus vulgaris* L. confirms its broad and versatile medicinal use.

In the “Antiviral agent” category, the mentioned species have a small number of reports (between 6 and 8), but some of them have high RFC values. Although antiviral use is less represented, the results confirm the universal cultural significance and role of species traditionally used by the Bulgarian population such as *Tilia* sp., *Matricaria chamomilla* L. and *Thymus vulgaris* L. *Zingiber officinale* Roscoe again shows moderate cultural significance.

### 2.4. Comparison of Results from the Ethnobotanical Survey in Bulgaria with Similar Studies Worldwide

Data from the conducted ethnobotanical survey were compared with results from analogous studies conducted in 14 countries from different continents: Europe (Turkey, Lithuania, Norway), Asia (Nepal, Vietnam, India, Thailand), South America (Colombia, Peru), and Africa (Algeria, Morocco, Ethiopia, Nigeria, Tunisia) [11,12,13,14,15,37,39,40,41,42,43,44,45,46,47].

In conducting the comparison, the fact was taken into account that a significant portion of medicinal plant species mentioned in foreign studies are not found in Bulgaria’s flora. Additionally, the possibility of variations in the biological activity of species used, conditioned by specific geographical and climatic conditions in different regions, was considered.

In view of this, the comparative analysis aims to present a more comprehensive picture of medicinal plants used on a global scale and to trace the effectiveness of phytotherapeutic approaches applied in different countries for the relief of respiratory symptoms similar to symptoms accompanying COVID-19 infection.

By number of reported medicinal plants, Bulgaria (45 species) ranks third globally, after Nepal (63 species) and Thailand (60 species).

The greatest number of matches regarding the species composition of cited medicinal plants in Bulgaria and abroad is with Turkey (15 species), Algeria (14 species), Colombia (13 species), and Lithuania (11 species) (Table 8).

Comparative analysis of data shows that the following species are most frequently cited in ethnobotanical studies worldwide: *Zingiber officinale* Roscoe—mentioned in 14 countries, *Mentha* sp.—mentioned in 10 countries, *Citrus x limon* (L.) Osbeck—mentioned in 9 countries, *Allium sativum* L.—mentioned in 9 countries, and *Cinnamomum* sp.—mentioned in 8 countries.

#### 2.4.1. Similarity Analysis by Use Categories

To evaluate the extent of overlap in ethnomedicinal knowledge between Bulgaria and other countries, a comparative analysis by use categories of medicinal plants was conducted, applying the Jaccard coefficient (JC) for quantitative assessment of similarity.

Table 9 presents the comparison between Bulgaria and the countries included in the analysis with respect to the similarity in medicinal plant species, distributed across use categories.

The highest values of the Jaccard coefficient are observed in the following use categories: “Headache” with Turkey (JC = 0.400), “Antiviral action” with Algeria (JC = 0.290), “Fatigue” with Lithuania (JC = 0.278), and “Respiratory problems” with Lithuania (JC = 0.238).

These data show the greatest similarity in ethnomedicinal practices between Bulgaria and the respective countries.

The analysis of medicinal plant species based on the reported use categories in Bulgaria and reveals taxa with internationally exceptional polyfunctionality: *Mentha* sp.—11 use categories, *Matricaria chamomilla* L.—9 use categories, *Thymus vulgaris* L.—9 use categories, *Tilia* sp.—7 use categories, *Allium sativum* L.—6 use categories, *Zingiber officinale* Roscoe—6 use categories and *Salvia officinalis* L.—5 use categories.

The high frequency of use of these species in multiple categories and countries supports the scientific expediency of in-depth studies of their biologically active potential. These data correlate with phytochemical and pharmacological studies initiated on a global scale of the indicated species as a promising source of biologically active substances in combating COVID-19 infection.

Regarding reported use categories domestically and abroad, analysis shows that foreign studies indicate additional categories for which there are no data specified in the Bulgarian study.

For example, for *Mentha* sp. in the Bulgarian study, 13 use categories are documented, supplemented by 4 more categories reported in international studies. The greatest match with international data is observed in the categories: “Cough” (four countries), “Antiviral agent” (three countries), “Sore throat” (three countries), and “Gastrointestinal tract problems” (three countries).

For *Zingiber officinale* Roscoe in the Bulgarian ethnobotanical study, eight use categories are documented. The most significant match with international data is in the categories: “Cough” (six countries), “Respiratory problems” (six countries), “Gastrointestinal tract problems” (five countries), “Sore throat” (five countries), “Antiviral action” (four countries), and “Immunostimulant” (four countries). In international studies, an additional nine use categories are registered that are not mentioned in the Bulgarian study.

For *Allium sativum* L., with six reported use categories in Bulgaria, the highest match with international studies is established in the categories: “Cough” (five countries), “Fatigue” (four countries), “Prevention of complications” (four countries), and “Fever” (four countries). In international studies, an additional seven use categories are identified.

*Citrus x limon* (L.) Osbeck, of a total of five reported use categories in Bulgaria, the strongest match with international data is in the categories “Cough” (three countries), and “Immunostimulant” (three countries). International studies report 11 additional use categories.

For *Cinnamomum* sp. in Bulgaria, only two use categories are reported: “Runny nose and loss of smell” (three countries) and “Cough” (two countries). International studies report an additional 12 use categories.

The significant number of additional uses not documented in the Bulgarian ethnobotanical study for species, such as *Citrus x limon* (L.) Osbeck, *Cinnamomum* sp. and *Zingiber officinale* Rescoe., is explained by the fact that these are foreign species whose use is culturally introduced and not traditionally integrated into Bulgarian folk medicine.

Based on the conducted comparative analysis, the following groups of medicinal plants can be prioritized: (1) plants with high ethnobotanical indicators in Bulgaria (highest citation frequency, broadest application spectrum, with highest values of ethnobotanical indices); (2) plants for the treatment of most common symptoms similar to COVID-19’s accompanying symptoms (respiratory symptoms, fever, fatigue, gastrointestinal symptoms); and (3) plants with international validation (species used simultaneously in Bulgaria and at least five other countries, species with high Jaccard coefficient values (JC > 0.20), and species mentioned in multiple use categories).

Based on integrated analysis, the following species are of highest scientific interest: *Mentha* sp. (11 use categories, 10 countries), *Zingiber officinale* Roscoe (6 use categories, 14 countries), *Matricaria chamomilla* L. (9 use categories, 10 countries), and *Thymus vulgaris* L. (9 use categories, 4 countries).

High priority is also distinguished for the species *Tilia* sp. (7 use categories, 2 countries), *Allium sativum* L. (6 use categories, 9 countries), *Salvia officinalis* L. (5 use categories, 4 countries) and *Citrus x limon* (L.) Osbeck (5 use categories, 9 countries).

Comparative analysis illustrates that systematic consolidation and analysis of ethnobotanical data from different parts of the world creates a solid foundation for targeted selection of promising medicinal plants.

During the COVID-19 pandemic, *Thymus vulgaris* L., *Matricaria chamomilla* L., *Tilia* sp., *Mentha* sp., *Sideritis scardica* Griseb, *Zingiber officinale* Roscoe, and *Citrus x limon* (L.) Osbeck form the core of global ethnomedicinal practice.

#### 2.4.2. Culturally Significant Plants

Based on the summarized, analyzed and quantitatively evaluated ethnobotanical information, the following plant species were identified as having significant sociocultural importance for the Bulgarian population *Thymus vulgaris* L., *Matricaria chamomilla* L., *Tilia* sp., *Mentha* sp., *Sideritis scardica* Griseb, *Zingiber officinale* Roscoe and *Citrus x limon* (L.) Osbeck.

## 3. Materials and Methods

### 3.1. Ethnobotanical Study and Data Collection

This ethnobotanical study was conducted from July 2022 to September 2023 and represents part of a comprehensive ethnobotanical investigation of folk knowledge about medicinal plants used for COVID-19 prevention and treatment among the population of the Republic of Bulgaria.

The study included 513 people from different settlements across the territory of the Republic of Bulgaria with different demographic indicators—gender, age, education, employment status.

Informants were randomly selected. After preliminary familiarization with the purpose and methods of the study, written informed consent was obtained from each participant.

Ethnobotanical information was collected using a pre-prepared online survey in the form of a Google form.

The survey was distributed via a link, directly by invitation from the researchers, through their contacts, friends and social media groups—using the respondent method and additional distribution on the “snowball” principle—from some respondents to others. The questionnaire includes questions aimed at collecting data regarding the demographic profile of participants, as well as questions gathering detailed ethnobotanical information concerning the use of medicinal plants for influencing respiratory symptoms similar to those accompanying COVID-19—local name, plant part, types of symptoms, form of application, treatment outcome, and source of information.

The study was approved by the Research Ethics Committee of the Medical University of Varna (№ 118/23.06.2022).

Data collected through the online survey underwent a validation process to ensure their reliability and consistency. Responses were reviewed for completeness of provided information, and incomplete or incorrect records were excluded from the analysis. Information about the use of specified medicinal plants was compared with available data from publications in the field of ethnobotany to establish correspondence or identify potentially new records.

The online survey methodology used in this study, although allowing access to a geographically diverse sample during pandemic restrictions, possesses several inherent limitations related to plant identification and survey design.

Reliance on local folk names provided by respondents without visual confirmation or herbarium specimens introduces potential taxonomic inaccuracies. Although we cross-referenced reported local names with established botanical literature for Bulgaria, some misidentifications may have occurred. Future studies should include direct methods for botanical verification.

The survey design does not allow for the collection of sufficiently extensive and evenly distributed data related to the regional origin or ethnic affiliation of respondents. This limits the ability to analyze geographical patterns of plant use and document differences between individual cultural communities in Bulgaria.

The methodological limitations indicate that online ethnobotanical surveys play a complementary role and primarily serve preliminarily to outline general trends, while preserving the leading significance of traditional field methods for comprehensive documentation of folk knowledge and practices.

### 3.2. Socio-Demographic Characteristics of Survey Respondents

Analysis of the territorial distribution of the studied sample of 513 survey respondents shows that they are located in 35 settlements in Bulgaria. The largest share consists of participants from the city of Varna (256; 52.7%), followed by participants from the cities of Pernik (41; 8.4%), Sofia (36; 7.4%), Burgas (28; 5.8%), and Dobrich (21; 4.3%). The remaining settlements are represented with smaller shares below 3%.

Regional distribution shows a clear predominance of northeastern Bulgaria (Varna, Dobrich, Silistra, Razgrad, Ruse, Shumen), with a significant number of participants, in which one or two are from small settlements from various geographical regions of the country.

Respondents are over 18 years of age, with the predominant age category being 18–30, whose share is approximately one half—46.8% of the total number of respondents.

The share of male respondents is twice as large as that of women (67%).

The percentage of survey participants with secondary and higher education is approximately equal (49.3% and 47%).

Regarding social status, the share of students (37.8%) together with that of employed persons (48.3%) constitute the larger part of respondents—86.1% (Table 10).

### 3.3. Quantitative Processing and Analysis of Collected Ethnobotanical Information

Various ethnobotanical indicators were used in the collection and analysis of the ethnobotanical data obtained in the study, allowing for quantitative and qualitative assessment of the traditional knowledge of surveyed participants regarding the use of medicinal plants for COVID-19 prevention and treatment.

The use of ethnobotanical indicators allows for the identification of widely popular and culturally significant medicinal species for a given local community, differentiation of potentially effective species for further pharmacognostic and pharmacological research, comparison between different ethnobotanical regions or groups, as well as tracking the stability of traditional knowledge and its integration with modern conventional medicine [38,48,49].

The following ethnobotanical indicators were calculated.

#### 3.3.1. Relative Frequency of Citation (RFC)

RFC is used to assess the popularity of plants within a given community. It shows how often all informants, regardless of the type of use, mention a given medicinal plant. It was calculated using the standard method according to the formula:

$$RFC=\frac{FC}{N}\;,$$

where FC is the number of informants mentioning the use of species and N is the total number of informants participating in the study who applied herbal treatment.

RFC values range from 0 to 1. They are based on the percentage of informants citing a particular medicinal species, taking into account its degree of popularity in the traditional knowledge of a given community [16,31,37,49,50].

#### 3.3.2. Use Value Index by Species (UV) [13,32,49]

UV is an ethnobotanical indicator that evaluates the relative importance or popularity of a given medicinal plant based on the frequency of mention by informants. It shows how widely and frequently a particular plant is used in a given community, regardless of what medicinal purposes.

UV is calculated according to the formula:

$$UV=\frac{UR}{N},$$

where UR represents the total number of use reports of the specific species, and N is the total number of informants participating in the study who applied herbal treatment.

UR refers to the number of times a plant is cited or mentioned for treating a particular condition. Its values range from 0 upward, with no fixed upper limit. UVs close to 0 indicate that the plant is rarely mentioned and, therefore, has low cultural or medicinal significance. Values above 1 indicate that, on average, informants report more than one use for the species, reflecting greater cultural relevance. Higher UVs demonstrate more widespread use and greater confidence among respondents in the medicinal importance of the species.

#### 3.3.3. Informant Consensus Factor (FIC)

FIC provides information about informant consensus regarding the use of a particular plant species for a specific disease [38].

It allows for the assessment of variability in medicinal plant use and is a useful tool for determining the most commonly used medicinal species for a specific disease, which are of particular interest or so-called culturally significant in the search for bioactive compounds [38].

The calculated FIC coefficient values allow for determining how well the traditional use of cited medicinal species is defined for different use categories.

FIC evaluates the relationship between the number of use reports in each category minus the number of taxa used, divided by the number of use reports in each category minus 1 [50].

The factor is calculated according to the formula:

$$FIC=\frac{NUR-Nt}{NUR-1},$$

where NUR is the number of individual plant use reports for a particular category, and Nt is the total number of species used by all informants for this category.

Factor values range from 0 to 1. High FIC values (close to 1) indicate high consensus—when a high proportion of informants report one or several plant species for a particular use category. Low FIC values indicate that informants do not have consensus regarding the use of mentioned species for treatment within the disease category [38,51,52].

#### 3.3.4. Fidelity Level Index (FL)

FL is defined as the percentage of informants who indicate a given plant for a specific disease or category, relative to all who mention that plant for any purpose.

The FL value is calculated as a percentage using the formula:

$$FL\left(\%\right)=\frac{Np}{N},$$

where Np is a number of informants who indicate the plant for a specific disease, and N is the total number of informants participating in the study who applied herbal treatment [51].

A high FL value (80–100%) means that the plant is used specifically for a given disease, while a low FL value (below 50%) indicates that there are many different uses without a clearly dominant one [48,53].

#### 3.3.5. Jaccard Coefficient (JC) [54]

For quantitative assessment of the degree of similarity between medicinal species used in Bulgaria and abroad by use categories, the Jaccard coefficient (JC) was applied, calculated by the formula:

$$JC=\frac{C}{\left(A+B+C^{\prime }\right)},$$

where A is a number of attributes present in operational taxonomic unit A, B is a number of attributes present in operational taxonomic unit B, and C is a number of attributes present in A and B.

## 4. Conclusions

This study is the first of its kind in Bulgaria, which provides a sample of culturally significant medicinal plants:

*Thymus vulgaris* L., *Matricaria chamomilla* L., *Tilia* sp., *Mentha* sp., *Sideritis scardica* Griseb., *Zingiber officinale* Roscoe and *Citrus x limon* (L.) Osbeck. These plants are strongly connected to Bulgarian folk medicine and have been used for generations to relieve symptoms similar to those accompanying COVID-19.

The high FL and RFC values, combined with strong informant consensus (FIC > 0.70), testify to the stability of information flow and widespread distribution of folk knowledge regarding the use of these species among the Bulgarian population.

*Thymus vulgaris* L., *Matricaria chamomilla* L., *Tilia* sp., *Mentha* sp., *Sideritis scardica* Griseb., *Zingiber officinale* Roscoe and *Citrus x limon* (L.) Osbeck represent a promising group of plants for pharmacological validation, especially regarding their antiviral, immunomodulation and symptom-relieving properties.

The high frequency of use and the reported multifunctionality indicate the potential of these culturally significant species as a platform and priority targets for phytochemical and pharmacological research.

The integration of traditional knowledge with modern scientific methods represents a promising approach for the development of new phytotherapeutic strategies and antiviral agents to combat symptoms similar to those of COVID-19, as well as future viral pandemics.

## Figures and Tables

**Table 1:** Citation frequency of plant parts.

Plant Part	Number of Reports	Percentages %
Flowers	312	38.05
Herb	200	24.39
Leaves	119	14.51
Root	90	10.98
Fruit	71	8.66
Seeds	16	1.95
Bulbs	10	1.22
Bark	2	0.24
Total	820	100

**Table 2:** Forms of application.

Forms of Application	Number of Reports	Percentages %
Decoction	394	48.05
Infusion	212	25.85
Fresh plant material	122	14.88
Tincture	44	5.36
Syrup	24	2.93
Essential oil	24	2.93
Total	820	100

**Table 3:** Method of procurement.

Method of Procurement	Number of Reports	Percentages %
Nature	340	41.46
Market	301	36.71
Pharmacy	159	19.39
Garden	20	2.44
Total	820	100

**Table 4:** Sources of ethnobotanical information specified by survey respondents.

Sources of Ethnobotanical Information	Number of Reports	Percentages %
Elderly people	275	33.54
Relatives and acquaintances	224	27.32
Specialized literature	139	16.95
Internet and media	130	15.85
Health professionals	37	4.51
Personal experience	15	1.83
Total	820	100

**Table 5:** Medicinal plants used during COVID-19 in Bulgaria.

Family	Scientific Name	Local Name (in English)	Local Name (in Bugaria)	Plant Part	Form of Use	Number of Respondents Mentioning the Plant	RFC	UV
Adoxaceae	*Sambucus ebulus* L.	Dwarf elder	Trevist baz	Flower	Infusion Syrup	5	0.2512	0.0258
Adoxaceae	*Sambucus nigra* L.	Elder	Darvesen baz	Fruit Leaves	Infusion Decoction	3	0.0154	0.0515
Amaryllidaceae	*Allium cepa* L.	Onion	Kromid luk	Bulbs	Decoction	1	0.0052	0.103
Amaryllidaceae	*Allium sativum* L.	Garlic	Chesan	Bulbs	In statu recenti	3	0.0154	0.0412
Anacardiaceae	*Cotinus coggygria* Scop.	Smoke tree	Smradlika	Leaves	Decoction	4	0.0206	0.0309
Apiaceae	*Pimpinella anisum* L.	Anise	Anason	Fruit	Aetheroleum	1	0.0052	0.0052
Apiaceae	*Apium graveolens* L.	Celery	Tselina	Seeds	In statu recent	1	0.0052	0.0052
Asteraceae	*Matricaria* chamomilla L.	Chamomile	Laika	Flower	Infusion Syrup	80	0.4124	0.7526
Asteraceae	*Tagetes erecta* L. *	Marigold	Turta	Flower	Infusion Syrup	2	0.0104	0.0155
Asteraceae	*Tussilago farfara* L.	Coltsfoot	Podbel	Leaves	Infusion Decoction	5	0.0258	0.0464
Asteraceae	*Echinacea* *purpurea* (L.) Moench*	Echinacea	Ehinatseya	Flower	Infusion Syrup	4	0.0206	0.0309
Asteraceae	*Achillea millefolium* L.	Yarrow	Byal ravnets	Flower	Infusion Syrup	1	0.0104	0.0309
Asteraceae	*Calendula officinalis* L.	Calendula	Neven	Flower	Infusion Syrup	1	0.0052	0.0052
Brassicaceae	*Sinapis nigra* L.	Black mustard	Cheren sinap	Seeds	In statu recenti	5	0.0258	0.0361
Brassicaceae	*Armoracia rusticana* G. Gaertn., B. Mey. & Scherb. *	Horseradish	Hryan	Root	In statu recenti	1	0.0052	0.0052
Brassicaceae	*Sinapis alba* L.	White mustard	Byal sinap	Seeds	Decoction	3	0.0154	0.0206
Clusiaceae	*Hypericum perforatum* L.	St. John’s wort	Zhalt kantarion	Herb	Infusion Decoction	7	0.0360	0.1031
Fabaceae	*Glycyrrhiza glabra* L.	Licorice	Sladak koren	Root	Infusion	2	0.0104	0.0206
Juglandaceae	*Juglans regia* L.	Walnut	Oreh	Fruit	Decoction In statu recenti	2	0.0104	0.0206
Lamiacea	*Clinopodium vulgare* L.	Wild basil	Koteshka stapka	Leaves	Tincture	3	0.0154	0.0258
Lamiaceae	*Mentha* sp.	Mint	Menta	Leaves	Decoction Infusion Tincture	39	0.2011	0.3660
Lamiaceae	*Thymus vulgaris* L.	Thyme	Mashterka	Herb	Decoction Infusion Tincture	59	0.3041	0.5670
Lamiaceae	*Sideritis scardica* Griseb.	Mountain tea	Mursalski chai	Flower	Infusion Syrup	24	0.1237	0.2010
Lamiaceae	*Salvia officinalis* L.	Sage	Gradinski chai	Leaves	Infusion Infusion	6	0.0310	0.0464
Lamiaceae	*Melissa officinalis* L.	Lemon balm	Matochina	Leaves	Infusion	8	0.0412	0.0619
Lamiaceae	*Origanum vulgare* L.	Oregano	Rigan	Leaves	Decoction	9	0.0464	0.0876
Lamiaceae	*Rosmarinus officinalis* L.	Rosemary	Rozmarin	Leaves	Infusion	1	0.0052	0.0052
Lauraceae	*Laurus nobilis* L.	Bay laurel	Dafinovo darvo	Leaves	Infusion	2	0.0104	0.0103
Lauraceae	*Cinnamomum verum* J.Presl *	Cinnamon	Kanela	Bark	In statu recenti	2	0.0104	0.0103
Linaceae	*Linum usitatissimum* L.	Flax	Kulturnen len	Seeds	In statu recenti	1	0.0052	0.0052
Malvaceae	*Tilia* sp.	Linden	Lipa	Flower	Infusion Syrup	72	0.3711	0.7165
Malvaceae	*Hibiscus sabdariffa* L. *	Hibiscus	Hibiskus	Flower	Infusion Syrup	1	0.0052	0.0103
Moraceae	*Ficus carica* L.	Fig	Smokinya	Leaves	Decoction	2	0.0104	0.0103
Pinaceae	*Pinus sylvestris* L.	Pine	Bor	Leaves	Decoction	1	0.0052	0.0103
Plantaginaceae	*Plantago* sp.	Plantain	Zhivovlek	Leaves	Decoction	2	0.0104	0.0108
Ranunculaceae	*Nigella sativa* L.	Black cumin	Cheren kimon	Seeds	Aetheroleum	1	0.0052	0.0155
Rosaceae	*Filipendula ulmaria* (L.) Maxim.	Meadowsweet	Blaten tazhnik	Flower	Infusion Syrup	1	0.0052	0.0206
Rosaceae	*Aronia melanocarpa* (Mich.) Elliott *	Chokeberry	Aronia	Fruit	Infusion	1	0.0052	0,.052
Rosaceae	*Rosa canina* L.	Dog rose	Shipka	Fruit	Decoction Infusion	25	0.1289	0.1392
Rosaceae	*Prunus spinosa* L.	Blackthorn	Tranka	Fruit	Infusion	4	0.0206	0.0206
Rutaceae	*Citrus x limon* (L.) Obeck *	Lemon	Limon	Fruit	In statu recent	29	0.1495	0.1289
Solanaceae	*Atropa belladona* L.	Belladonna	Beladona	Fruit	Decoction	1	0.0052	0.0052
Urticaceae	*Urtica dioica* L.	Nettle	Kopriva	Leaves	Decoction	2	0.0104	0.0155
Zingiberaceae	*Zingiber officinale* Roscoe *	Ginger	Dzhindzhifil	Radix	Decoction In statu recenti	48	0.2475	0.4381
Zygophyllaceae	*Tribulus terrestris* L.	Puncture vine	Babini zabi	Herb	Decoction	1	0.0052	0.0052

* foreign medicinal plants.

**Table 6:** Informant consensus factor (FIC) for different use categories.

Use Category	Number of Taxa (n)	Number of Use Reports (n)	FIC
Cough	24	189	0.8777
Prevention of complications	26	168	0.8503
Sore throat	18	101	0.8300
Antiviral agent	9	39	0.7895
Immunostimulant	21	79	0.7436
Prophylaxis	17	59	0.7241
Treatment of overall symptoms	22	68	0.6866
Respiratory problems	15	43	0.6667
Runny nose and loss of smell	9	24	0.6522
Headache	4	9	0.6250
Fever	7	14	0.5384
Sedative	4	6	0.5000
Fatigue	10	18	0.4706

**Table 7:** Fidelity level (FL) and cultural significance of medicinal plants cited in the survey, distributed by use categories.

Medicinal Plant	Number of Reports	FL %	RFC	Cultural Significance
Category “Cough”				
*Thymus vulgaris* L.	32	54	0.30	High cultural significance
*Tilia* sp.	34	47	0.37	High cultural significance
*Sideritis scardica* Griseb.	10	42	0.12	Specific cultural significance
*Zingiber officinale* Roscoe	18	37	0.25	Universal cultural significance
*Matricaria chamomilla* L.	28	35	0.41	Universal cultural significance
*Mentha* sp.	13	33	0.20	Universal cultural significance
Category “Prevention of complications”				
*Tilia* sp.	34	47	0.37	High cultural significance
*Sideritis scardica* Griseb.	11	46	0.12	Specific cultural significance
*Matricaria chamomilla* L.	36	45	0.41	Moderate cultural significance
*Thymus vulgaris* L.	21	36	0.30	Moderate cultural significance
*Zingiber officinale* Roscoe	18	37	0.25	Established foreign species with specific local use
*Mentha* sp.	10	26	0.20	Universal cultural significance
Category “Sore throat”				
*Tilia* sp.	28	39	0.37	High cultural significance
*Matricaria chamomilla* L.	23	29	0.41	Universal cultural significance
Category “Immunostimulant”				
*Zingiber officinale* Roscoe	12	25	0.25	Moderate cultural significance
*Citrus x limon* (L.) Osbeck	9	31	0.15	Specific cultural significance
*Tilia* sp.	9	13	0.37	Universal cultural significance
*Thymus vulgaris* L.	7	12	0.64	Universal cultural significance
Category “Prophylaxis”				
*Zingiber officinale* Roscoe	12	25	0.25	Moderate cultural significance
*Thymus vulgaris* L.	7	12	0.64	Universal cultural significance
*Mentha* sp.	6	15	0.20	Moderate cultural significance
*Citrus x limon* (L.) Osbeck	6	21	0.15	Specific cultural significance
Category “Antiviral agent”				
*Tilia* sp.	7	10	0.37	Universal cultural significance
*Matricaria chamomilla* L.	6	8	0.41	Universal cultural significance
*Thymus vulgaris* L.	8	14	0.64	Universal cultural significance
*Zingiber officinale* Roscoe	6	13	0.25	Moderate cultural significance

Note: High cultural significance—widespread, specific traditional use (high FL + high RFC); moderate cultural significance—established but limited to several applications traditional use (medium FL + medium RFC); specific cultural significance—locally specific, traditionally established medicinal use (high FL + low RFC); universal cultural significance—widely used in traditional medicine plant with many medicinal applications, without being narrowly specialized (low FL + high RFC).

**Table 8:** Number of medicinal plant species reported in the ethnobotanical survey and overlapping species between Bulgaria and selected countries.

Country	Number of Reported Medicinal Plants	Number of Common Medicinal Plants	References
Nepal	63	9	[12]
Thailand	60	2	[43]
Turkey	35	15	[37,39]
Columbia	33	13	[13]
Nigeria	27	3	[46]
Algeria	22	14	[15]
Morocco	22	8	[44]
India	18	5	[42]
Peru	17	9	[14]
Tunisia	15	7	[47]
Lithuania	14	11	[11]
Vietnam	13	3	[41]
Ethiopia	12	6	[45]
Norway	8	3	[40]

**Table 9:** Comparison of shared medicinal plant species between Bulgaria and other countries by therapeutic category and Jaccard index.

Category	Country	Jaccard Index	Shared Species
Cough	Lithuania [11]	0.19	*Matricaria chamomilla* L., *Mentha* sp., *Thymus vulgaris* L., *Salvia* *officinalis* L., *Tilia* sp., *Urtica dioica* L.
	Peru [14]	0.18	*Matricaria chamomilla* L., *Thymus* *vulgaris* L., *Salvia officinalis* L., *Melissa* *officinalis* L., *Origanum* *vulgare* L., *Zingiber officinale* Roscoe
Immunostimulant	Nepal [12]	0.12	*Allium cepa* L., *Ocimum tenuiflorum* L., *Nigella sativa* L., *Citrus aurantifolia* (Christ.) Swingle, *Zingiber officinale* Roscoe
Prevention of complications	Algeria [15]	0.19	*Mentha* sp., *Thymus vulgaris* L., *Citrus x limon* (L.) Osbeck., *Zingiber officinale* Roscoe
	Peru [14]	0.17	*Matricaria chamomilla* L., *Allium cepa* L., *Origanum vulgare* L., *Zingiber officinale* Roscoe
Antiviral agent	Algeria [15]	0.29	*Mentha* sp., *Thymus vulgaris* L., *Matricaria chamomilla* L., *Allium* *sativum* L., *Origanum vulgare* L., *Salvia officinalis* L., *Zingiber officinale* Roscoe
	Nepal [12]	0.13	*Mentha* sp., *Zingiber officinale* Roscoe
	Tunisia [47]	0.11	*Allium sativum* L., *Zingiber officinale* Roscoe
	India [42]	0.09	*Mentha* sp., *Zingiber officinale* Roscoe
Sore throat	Turkey [37,39]	0.23,	*Mentha* sp., *Melissa officinalis* L., *Rosa canina* L., *Salvia officinalis* L., *Sideritis scardica* Griseb., *Thymus vulgaris* L., *Tilia* sp.
	Lithuania [11]	0.24	*Matricaria chamomilla* L., *Mentha* sp., *Salvia officinalis* L., *Thymus vulgaris* L., *Tilia* sp., *Urtica dioica* L.
	Peru [14]	0.24	*Matricaria chamomilla* L., *Mentha* sp., *Salvia officinalis* L., *Thymus vulgaris* L., *Tilia* sp., *Urtica dioica* L.
Prevention	Algeria [15]	0.20	*Mentha* sp., *Thymus vulgaris* L., *Citrus x limon* (L.) Osbeck., *Zingiber officinale* Roscoe
Runny nose and loss of smell	Lithuania [11]	0.16	*Matricaria chamomilla* L., *Mentha* sp. L., *Thymus vulgaris* L., *Tilia* sp.
	Tunisia [47]	0.13	*Eucalyptus tereticornis* L’Hér., *Cinnamomum* sp., *Mentha* sp.
Fatigue	Lithuania [11]	0.28	*Allium sativum* L., *Matricaria* *chamomilla* L., *Mentha* sp., *Thymus* *vulgaris* L., *Tilia* sp.
Fever	Peru [14]	0.17	*Allium sativum* L., *Matricaria* *chamomilla* L.
	Tunisia [47]	0.10	*Allium sativum* L., *Mentha* sp.
Respiratory problems	Lithuania [11]	0.24	*Allium sativum* L., *Matricaria* *chamomilla* L., *Mentha* sp., *Salvia* *officinalis* L., *Thymus vulgaris* L., *Tilia* sp.
	Turkey [37,39]	0.22	*Echinacea* sp., *Rosa canina* L., *Salvia officinalis* L., *Thymus vulgaris* L., *Zingiber officinale* Roscoe
	Tunisia [47]	0.13	*Mentha* sp., *Nigella sativa* L. *Zingiber officinale* Roscoe
Overall improvement	Lithuania [11]	0.20	*Calendula officinalis* L., *Matricaria* *chamomilla* L., *Mentha* sp., *Salvia* *officinalis* L., *Thymus vulgaris* L., *Tilia* sp.
Headache	Turkey [37,39]	0.40	*Mentha* sp., *Tilia* sp.
	Tunisia [47]	0.13	*Mentha* sp., *Nigella sativa* L.

No overlap was observed in the category “Sedative” between the data from the Bulgarian ethnobotanical study and the international sources.

**Table 10:** Socio-demographic characteristics of survey participants.

Socio-Demographic Characteristics	Categories	Frequency %
Gender	Women	32.3
	Men	67.7
Age	18–30	46.8
	31–40	8.6
	41–50	22.1
	51–65	12.9
	65+	9.6
Education	Primary	2.3
	Secondary	49.3
	Higher	47
Social employment	Student	37.8
	Employed	48.3
	Unemployed	2
	Pensioner	10.2

## Data Availability

The original contributions presented in this study are included in the article. Further inquiries can be directed to the corresponding author.
